# Numerical modeling of dielectric barrier discharge actuators based on the properties of low-frequency plasmons

**DOI:** 10.1038/s41598-022-14370-z

**Published:** 2022-06-20

**Authors:** D. Soltani Tehrani, G. R. Abdizadeh, S. Noori

**Affiliations:** Aerospace Engineering Department, Amirkabir University of Technology, Tehran, Iran

**Keywords:** Aerospace engineering, Mechanical engineering, Fluid dynamics, Plasma physics

## Abstract

Electrohydrodynamic flow control systems have proven to be among the most promising flow control strategies within previous decades. Several methods for efficient evaluation and description of the effect of such systems are indeed available. Yet, due to these systems’ critical role in various applications, possible improvements are still investigated. A new phenomenological model is presented for the simulation of the plasma actuators based on the electrodynamic properties of low-frequency plasmons. The model simulates the plasmonic region as a dispersive medium. This dissipated energy is added to the flow by introducing a high-pressure region, calculated in terms of local body force vectors, requiring the distribution of the electric field and the polarization field. The model determines the electric field for the computation of the body force vector based on the Poisson equation and implements the simplified Lorentz model for the polarization field. To fully explore the performance of the presented model, an experiment has been conducted providing a comparison between the observed effect of plasma actuators on the fluid flow with the results predicted by the model. The model is then validated based on the results of other distinct experiments and exempted numerical models, based on the exchanging momentum with the ambient neutrally charged fluid, demonstrating that the model has improved adaptability and self-adjusting capability compared to the available models.

## Introduction

Electrohydrodynamic flow control systems have proven to be among the most promising flow control strategies within previous decades. Among these systems, plasma actuators have been confirmed to be effective in a wide variety of applications, including flow control purposes, photonics and optoelectronics, food processing technologies, cancer treatment, and biotechnology^1–6^. Literature shows a strong background, investigating and improving the applicability and effectiveness of different flow control methods in several application fields^7–16^. However, a thorough development and testing process is required to incorporate the resulting systems into actual applications. Numerical simulations have traditionally attempted to give advanced algorithms for designing, simulating, and comprehending complicated flow control systems, as the experimental approach requires several costly and time-consuming trial-and-error iterations. Several methods for efficient evaluation and description of the effect of dielectric barrier discharge (DBD) systems are currently available through the literature. However, due to these systems’ critical role in many flow control problems, possible improvements are always worth investigating, and an improved algorithm is always welcome.

There are currently three categories of models for simulating plasma actuators; models based on fundamental principles^17–21^, empirical models^22,23^, and phenomenological models^24–28^. In order to form frameworks for first-principles-based methodologies, models in the first category attempt to reproduce the physical mechanisms of a plasma actuator, both from the hydrodynamical side^20,21,29,30^ and from the plasma side^17–19^. Therefore, these models are required to consider transport equations for both charged and neutral species, as well as the Poisson equation for the electric field and the Navier-Stokes equations. These models are of more accuracy while requiring notable computational cost and time. The second category attempts to impose an accurate description of the induced body force of plasma actuators in the momentum equations. These models consider developing practical modeling tools for DBD actuators for fast design, control, and optimization purposes. The final category of models uses simplified sets of differential equations, resulting in less computationally demanding simulations while considering the contributing physics with simplifications and maintaining an acceptable level of accuracy. In recent years, there has been much research on plasma actuators. In the first place, the current work reviews some of the exempted past experimental and numerical research on plasma actuators, then discusses thoughts and fundamentals in order to better understand the underlying physical mechanisms of the actuator’s interaction with the flow and to develop a new practical methodology for simulating plasma actuators. Based on the descriptions above regarding the different categories of models for simulating plasma actuator, this study is then going to provide a phenomenological model for the simulation of low-frequency plasma actuators. In what continues, specifically, phenomenological models will be studied.

**Figure 1:**
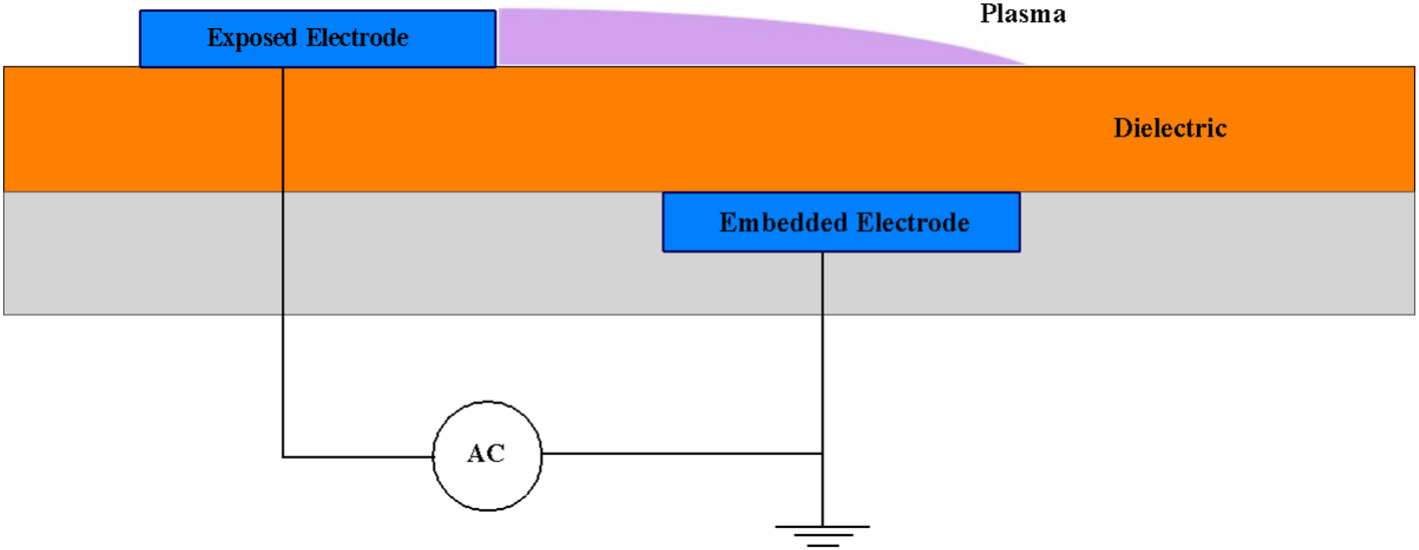
DBD plasma actuator schematic.

The plasma actuators are comprised of two electrodes separated by a dielectric substance, as shown in Fig. 1. When it comes to DBD plasma actuators, they can be classified as self-sufficient compared to plasma actuators that need an external source of creating charged particles that can be impacted by an electric or magnetic field.

The self-contained plasma actuators generate their own electric field and charged particles to apply electric force on them. The air around the electrodes ionizes weakly when an AC voltage is applied to them. Differences between the phenomenological models come into a discussion based on ways of characterizing and then implementing the consequences of this weakly ionized medium, considered as the plasmonic medium. Shyy et al.^26^ characterized the exterior flow effects of the plasma actuators as a time-averaged mean body force spread in a triangular area above the embedded electrode. Suzen and Huang^27,31^ proposed a model using the plasma formulation of Enloe et al.^32^ based on the experimental data, reducing the Maxwell equations, considering the plasma formation as a quasi-steady process, and ignoring magnetic forces. In the Navier-Stokes equations, the induced body force was introduced as a source term. The charge distribution over the dielectric surface was assumed to have a 1-D Gaussian distribution based on the experimental data^33^. Many improvements to this formulation have been presented in the literature^28,34–37^. Orlov and Corke^38,39^ used a lumped parameter model to simulate the plasma actuator effects. Different versions of the electric force obtained based on the calculated charges and electric fields from different models has been employed to compute the flow actuation effect across the literature^21,38–42^.

**Table 1:** Summary of the features and contributions of the phenomenological models that are similar to the one presented.

References	Contribution	Main features
Shyy et al.^26^	Models the influence of plasma actuators on external flow as a time-averaged mean body force spread in a triangular area above the implanted electrode; linearizes the electric field	Based on experimental results, uses a predetermined volume charge density and plasma volume
Suzen et al.^27,31^	Calculates the body force by solving the Poisson equation and then adding the resultant body force to the Navier-Stokes equations as a source term	Assumes a Gaussian charge distribution and introduces maximum charge density and standard deviation parameters that will be regulated based on experimental results
Orlov et al.^38,39^	To find the actuator body force vector, which is utilized to govern the amount of energy injected into the flow, solves a time-dependent charge distribution equation to provide boundary conditions for the electric field equation	No need to make a prior assumption of charge density boundary condition while solving; adds an extra equation to regulate the injected energy into the fluid flow
Abdollahzedeh et al.^28^	The model of Suzen and Huang has been improved; constructs a simple model for plasma discharge and its effect on flow, based on scaling the thrust produced by DBD plasma actuators. To estimate and simulate the body force distribution created by the plasma actuator, introduces scales into a basic phenomenological model	Adds an extra equation to scale the thrust of the actuator; properly forecasts the nonlinear relationship between thrust produced and applied voltage
Omidi et al.^34,35^	Suzen and Huang’s model has been improved; adds an empirical relationship to relate variations in frequency to the Debye length as one of the characterizing factors	Changes the boundary conditions in Suzen and Huang’s model; best suited for optimization, particularly for turbine airfoil designs
Bouchmal^36^	Suzen and Huang’s model has been improved; includes a relationship that incorporates changes in the voltage to the Debye length as well as changes in the standard deviation of the Gaussian charge distribution boundary condition	Provides relations for the prediction of characterizing parameters of Suzen and Huang’s model

A literature review reports that much effort has been expended on simulating the plasma actuation effect on fluid flow as accurately as feasible while keeping the computational cost near the optimum. However, the previous works introduce the plasmonic region as a volume charge density distribution, being exposed to an electric field that produces and transfers momentum into the fluid flow. These models require characterizing parameters to be regulated based on the experiments due to changes in the actuator’s excitation features or configuration. The concerns appear when new fields of application for these controlling devices face scalability, layout design, and optimization challenges, and the existing models fail to provide enough flexibility.

Despite the fact that computational investigation of the underlying physics of DBD actuators has been extremely difficult, treating the plasmonic region as an excited material based on its frequency responsive properties, rather than predicting a space charge density distribution for the region, leads to a modulable model. We aim to present a new numerical methodology for plasma actuator-based active flow control applications. In this innovative approach, the plasmonic zone is replicated using a practical material model, the Lorentz model^43^. As stated previously, this model goes under the category of phenomenological models. Table 1 summarizes the features of the previous phenomenological models that are near to our modeling perspective in order for a better comparison. The next section goes into the model’s development and implementation in detail. The electrodynamic computations for a single plasma actuator and computations of flow control in quiescent flow using a single plasma actuator are given in the “Results and discussion” section. Concluding remarks would come in the section titled “Conclusion”section. The final section titled “Method”section provides details of the conducted experiments.

## Development of the computational model

A DBD plasma actuator is mounted on the surface of any device, with one electrode exposed to the environment and the other embedded in the dielectric material below the surface (Fig. 1). The plasmonic zone is created when high-amplitude alternating voltage is delivered to the electrodes, which causes the air around them to ionize weakly. As previously mentioned, distinctions between phenomenological models are discussed based on methods of describing and subsequently applying the implications of this weakly ionized medium, referred to as the plasmonic medium.

To simplify the process of plasma actuation, we know from self-sufficient plasma devices that they generate the Lorentz force by providing themselves charged particles through ionization of the medium as well as their required electric field. While analyzing and estimating the electric field is rather straightforward in this context, there are three distinct methods for simulating the generation and distribution of charged particles.

The fundamental way to mimic the generation and distribution of charged particles is to simulate the exact molecular interaction and solving complicated transport equations for charged and neutral species. Another technique to approach this phenomena is macroscopically, by empirically or semi-empirically calculating the charge density distribution. This method may give an ideal model when the integral features of a system are important; nevertheless, for industrial applications, the integral parameters such as total lift force, total drag force, and total torque generated are critical. Models following the latter technique typically contain constants that must be determined experimentally for each DBD configuration and study case (e.g., in Shyy’s model, we must obtain the charge density of the plasma region from an experiment as well as having a fixed plasma region, or in Suzen’s model, we must tune the peak charge density or standard deviation of the charged particle normal distribution with the experiment).

This study, however, aims to answer a bigger question, whether we can provide tunability to the plasma actuation in terms of both the magnitude and the direction of the injected momentum vector, while still keeping the computational cost near optimum, compared to the first-principle-based models. In this regard, there is this need to develop a model that provides us with physics-based controlling parameters. This latter specification makes the model independent of the experiments conducted for each study case in order to determine the tuning parameters, as is the case with Suzen’s or the Shyy’s models.

With the above explanation, the challenge that arises is how to describe the plasmonic region in order for it to be self-configurable within the model. We propose in this study that we can represent the plasma region as if it were a medium that is excited at a certain frequency. As a consequence, the domain’s permittivity and permeability would alter. While permittivity and permeability are often described in terms of constant (frequency-independent) values, in fact, all material characteristics are frequency dependent. Numerous material models have been developed to characterize materials’ frequency response. The Lorentz model is one of the most well-known material models. It is developed from an analogy to the electron’s motion as a driven, damped harmonic oscillator. When restoring forces are negligible, a simplification of the Lorentz model yields the Drude model, which is employed in our modeling.

Assuming the plasma is quasi-neutral with ions too heavy to respond to electromagnetic field fluctuations, coupling between the electromagnetic response of the medium and the plasma occurs primarily through the electron current density. Due to the wave impedance of the medium in which an electromagnetic wave propagates,  focus is usually on how the electric field impacts electron motion in the presence of the nucleus, and hence the basic dipole moment of this system. Based on this behavior, models of the medium’s electric susceptibility and, as a result, permittivity have been developed. As said, one of the most popular material models is the Lorentz model, which represents the temporal reaction of a component of a medium’s polarization field to the same electric field component. According to the Lorentz model, a medium excited by an applied electromagnetic wave is defined by the created polarization field and consequently, the region’s permittivity. The plasma permittivity, or more accurately, the electromagnetic frequency, plasma frequency, and frequency of electron-neutral collisions, governs wave propagation, evanescence, or attenuation. When it comes to DBD plasma actuators, the characterizing permittivity of the plasmonic region becomes attenuating, introducing the region as a dispersive medium. In this model, this dispersed energy is expressed in terms of a volume body force. It is then incorporated into the Navier Stokes equations to mimic the energy transfer to the fluid flow.

### Body force formulation

The electrohydrodynamic (EHD) force is defined as,

1 $$\begin{aligned} \vec {f_b}=\rho _c\left( \vec {E\ }+\vec {V\ }\times \vec {B\ }\right) \end{aligned}$$

where $\vec {f_b}$, is the body force per unit volume, $\rho _c$, is net the charge density, $\vec {E}$, is the electric field intensity, $\vec {V\ }$, is the velocity vector, and $\vec {B\ }$, is the magnetic field.

Before getting into the details of the mathematical modelling of this study, let us provide two basic discussion. In this regard, what follows provide an overview of the electrodynamic analysis of a DBD actuator system. Afterwards, the Lorentz model is introduced to be used in later discussions.

#### Electrodynamic analysis of a DBD actuator system

In general, to explain the electrodynamic properties of any system the following four Maxwell equations are implemented:

2 $$\begin{aligned} curl\vec {H}= & {} \vec {j}+\frac{\vec {\partial D}}{\partial t} \end{aligned}$$

3 $$\begin{aligned} curl\vec {E}= & {} -\frac{\vec {\partial B}}{\partial t} \end{aligned}$$

4 $$\begin{aligned} div\vec {D}= & {} \rho _c \end{aligned}$$

5 $$\begin{aligned} div\vec {B}= & {} 0 \end{aligned}$$

where $\vec {H}$, is the magnetic field intensity, *j*, is the electric current, $\vec {D}$, the electric induction vector, representing the force induced to dielectric by the electric field. In addition, two constitutive relations are required to make the above four equations sufficient to permit a solution. These equations have usually been introduced in terms of the two material field vectors $\vec {P}$ and $\vec {M}$, the polarization density and the magnetization density,

6 $$\begin{aligned} \vec {D}= & {} \varepsilon _0 \vec {E} + \vec {P} \end{aligned}$$

7 $$\begin{aligned} \vec {B}= & {} \mu (\vec {H} + \vec {M}) \end{aligned}$$

with $\varepsilon _0$, and $\mu _0$ representing the free space permittivity and permeability, respectively. In DBD plasma actuators, the actuation process is studied based on the energy transfer form the ionized plasmonic region to the ambient flow. The fluid characteristic time scales for low-speed, incompressible fluid flow applications, which is the present study’s emphasis, are considerably larger than the operational plasma dynamics. In other words, as the electric field generation and ion rearrangement are much faster than the flow response, the energy generation part can be considered as a quasi-stable process, and the link between fluid and plasma physics may be safely treated in one direction, from plasma to the fluid flow. Therefore, the ions’ arrangement will be considered constant, and the current will be zero^21,38,40^. Also, all time-derivatives in the above equations become zero, and the only remaining equation will be what follows,

8 $$\begin{aligned} div\vec {D}= & {} \rho _c \end{aligned}$$

9 $$\begin{aligned} curl\vec {E}= & {} 0 \end{aligned}$$

10 $$\begin{aligned} \vec {D}= & {} \varepsilon _0 \vec {E} + \vec {P} \end{aligned}$$

Equation 9 yield that the gradient of a scalar potential can be used to calculate the electric field,

11 $$\begin{aligned} \vec {E}=-\vec {\nabla }\varphi \end{aligned}$$

The electric susceptibility is related to polarization and electric fields as,

12 $$\begin{aligned} \chi _{e}\left( \omega \right) =\frac{P_i\left( \omega \right) }{\varepsilon _0E_i\left( \omega \right) } \end{aligned}$$

Defining the effective permittivity of any medium as,

13 $$\begin{aligned} \varepsilon \left( \omega \right) =\varepsilon _0\left[ 1+\chi _e\right] \end{aligned}$$

with $\varepsilon _r$, as the relative permittivity of the medium, Eq. 10 yields,

14 $$\begin{aligned} \vec {D}=\varepsilon \vec {E} \end{aligned}$$

Gauss’s law then yields

15 $$\begin{aligned} \nabla \left( \varepsilon \nabla \varphi \right) =-\rho _c \end{aligned}$$

The above formulation suggests that, with having a known electric field intensity, one needs to describe the charge density distribution to result in the produced body force. Dissimilar to other approaches in the literature, we aim to go one step back to study the polarization field of a medium in response to its excitation with a particular frequency. As previously stated, in this study, we will use the Lorentz Oscillator model to follow the consequences of excitation on the medium’s electric permittivity. In what follows, the Lorentz model is presented and will be discussed further.

#### The Lorentz oscillator model

According to the Lorentz oscillator model, an electron is modeled as a driven damped harmonic oscillator. In this scenario, the electron is connected to the nucleus through a hypothetical spring with a spring constant of C. The driving force is the oscillating electric field. Although the source of the damping force is unknown, it exists to avoid unending oscillations when the driving force is at resonance. The goal of this model is to determine the electron’s velocity using Newton’s Second Law, from which formulae for the dipole moment, polarization, susceptibility, and dielectric constant may be derived.

Let us consider the driving oscillating electric field as $E = E_0 cos(-\omega t)$ (the minus in $cos(-\omega t)$ is to guarantee that it matches time dependence of a standard traveling electromagnetic wave). Describing the velocity-dependent damping force by the damping coefficient $\Gamma _L$, we would have,

16 $$\begin{aligned}&F_{net}=m\ddot{x} \end{aligned}$$

17 $$\begin{aligned}&F_{driving} + F_{restoring/spring} + F_{damping} = m\ddot{x} \end{aligned}$$

18 $$\begin{aligned}&q E_0 cos(-\omega t) - Cx - \Gamma _L m{\dot{x}} = m\ddot{x} \end{aligned}$$

Rearranging the latter equation, we would have,

19 $$\begin{aligned} \ddot{x} + \Gamma _L {\dot{x}} + \omega _0^2 x = \frac{qE}{m}\cos {\left( -\omega t\right) } \end{aligned}$$

with *m* representing the mass of the bulk of material under study, *q* the electric charge, and $\omega _0$ the Natural frequency of the system and $\omega _0 = \sqrt{C/m}$. For the purpose of this study, we need to formulate the Polarization field, based on the introduced Lorentz model. The polarization, *P*, is the dipole moment per volume. The complex dipole moment induced by an electron moving like what was explained in an atom, with the nucleus at the origin, stationary so it does not contribute to the dipole moment is given as,

20 $$\begin{aligned} \vec {p}=\sum q_ir_i \end{aligned}$$

If we assume that there are *n* electrons per volume, the polarization *P* is then derived from the following equation,

21 $$\begin{aligned} \ddot{P_i} + \Gamma _L \dot{P_i} + \omega _0^2 P_i = \varepsilon _0\chi _LE_i \end{aligned}$$

where $f_0 = \omega _0 / 2\pi$, represents the characteristic frequency of the restoring forces, and $\chi _L$ the coupling coefficient of the right-hand side driving term. The expression gives the frequency response, assuming the standard $exp(+j\omega t)$ time dependency, as,

22 $$\begin{aligned} P_i\left( \omega \right) =\frac{\varepsilon _0\chi _LE_i}{-\omega ^2+j\Gamma _L\omega +{\omega _0}^2} \end{aligned}$$

The response is resonant at the natural frequency with negligible losses $f_0$. From 12 The electric susceptibility of the Lorentz model would be,

23 $$\begin{aligned} \chi _{e,Lorentz}\left( \omega \right) =\frac{P_i\left( \omega \right) }{\varepsilon _0E_i\left( \omega \right) }=\frac{\chi _L}{-\omega ^2+j\Gamma _L\omega +{\omega _0}^2} \end{aligned}$$

The restoring force is insignificant in a lightly ionized region. As a result, the Drude model is obtained, represented as,

24 $$\begin{aligned} \chi _{e,Drude}\left( \omega \right) =\frac{\chi _D}{-\omega ^2+j\Gamma _L\omega } \end{aligned}$$

where the plasma frequency, $\omega _p=\sqrt{\frac{e^2n_e}{\varepsilon _0m_e}}$, generally represents the coupling coefficient, $\chi _D={\omega _p}^2$. The permittivity of the Lorentz-Drude model then comes as,

25 $$\begin{aligned} \varepsilon _{Lorentz-Drude}\left( \omega \right) =\varepsilon _0\left[ 1+\chi _{e,Lorentz-Drude}\right] \end{aligned}$$

It is critical to note that even in such a straightforward situation, the equation results in a complex permittivity, which results in complex wave vectors and refractive indices. This implies that permittivity and, hence, index of refraction are frequency dependent, implying dispersion. This dispersion plays the critical role in our proposed model.

#### Mathematical modelling

To proceed to study the particular issue of a DBD actuator structure, the very first question that emerges is, what do we precisely know about an operating DBD setup? From the boundary conditions of a DBD actuator setup, we know the voltage on the two electrodes, as well as the excitation frequency of the voltage between the two electrodes. We would have,

26 $$\begin{aligned} \varphi \left( t,\vec {r}\right) =\phi \left( \vec {r}\right) f\left( t\right) \end{aligned}$$

where $\varphi$ is the time-dependent electric potential, and $\phi$ the time-independent electric potential. Since the time dependency is caused solely by the boundary condition for the applied voltage at the exposed electrode, by applying a constant boundary condition, the related equations and voltage boundary conditions are rendered time-independent and solved. Note that we assume that this time-dependency of the boundary conditions is decoupled from the hydrodynamic features of the flow field. The following are the normalized parameters for 2-D coordinates:

27 $$\begin{aligned} \phi= & {} \frac{\varphi }{f\left( t\right) } \end{aligned}$$

28 $$\begin{aligned} \vec {E}= & {} \nabla \phi \ \end{aligned}$$

where *f*(*t*) is a function that represents the voltage’s waveform. Having the electric potential on the electrodes, we need to solve the governing integral equation below, to find the electric charge density distribution on the two electrodes that are responsible for the electric potential on the electrodes.

29 $$\begin{aligned} V_{applied}= & {} \int \rho \left( \vec {r}\right) G\left( \vec {r},\vec {r^\prime }\right) dr_1dr_2 \end{aligned}$$

where $V_{applied}$ is the applied voltage on the upper electrode. We are assuming that the lower electrode is set to be as ground. $G\left( \vec {r},\vec {r^\prime }\right)$ represents the Green’s Function of the medium that provides the medium’s response to a Dirac Delta source in the domain. With the electric charge distribution on the electrodes, the solution of the Eq. 15, results in the electric potential field around the DBD actuator setup. With the space electric potential field at hand, Eq. 28 results in the space electric intensity field around the DBD actuator system. As previously noted, plasma frequency is determined by electron density, $n_e$, and the charge of an electron, e, the free space permittivity, $\varepsilon _0$, and the electron mass, $m_e$. The electron density changes within the range of $10^{17}$–$10^{20}$ $m^{-3}$ based on the gas flow pressure. Knowing the excitation frequency, we can now implement the Eq. 25. Consequently, we can define the effective permittivity of the plasmonic medium as,

30 $$\begin{aligned} \varepsilon= & {} \varepsilon ^\prime +i\varepsilon ^{\prime \prime } \end{aligned}$$

The Lorentz-Drude model then results as below,

31 $$\begin{aligned} \varepsilon ^\prime= & {} \varepsilon _0 \end{aligned}$$

32 $$\begin{aligned} \varepsilon ^{\prime \prime }= & {} \varepsilon _0\chi _{e,Drude} \end{aligned}$$

Finding the divergence of the electric distance field from the Eqs. 10, and then 4, results in the space electric charge distribution around the DBD setup. To think backward to the problem at hand, this space electric charge distribution around the DBD setup is responsible for the previously found electric, electric potential and electric distance fields.

Now that we have both the space electric field and the space electric charge distribution, the resultant volume body force is expressed as,

33 $$\begin{aligned} {\vec {f}}_b\left( \vec {r}\right) =\rho \left( \vec {r}\right) \cdot \vec {E}\left( \vec {r}\right) \end{aligned}$$

### The numerical simulation method

For fluid flow modeling, the 2-D incompressible Reynolds Averaged Navier-Stokes (RANS) equations are utilized. It is anticipated that the bulk of the energy provided by the plasma actuator is mostly used to accelerate the fluid particles; thus, the amount contributing to fluid warming is deemed unimportant, and the flow field energy equation is neglected^44^. The following are the fundamental equations of momentum and mass conservation that were used for fluid flow simulation:

34 $$\begin{aligned} {\nabla }.{\vec {u}}= & {} 0 \end{aligned}$$

35 $$\begin{aligned} \left( \vec {u}.\nabla \right) \vec {u}= & {} -\frac{1}{\rho }\nabla P+\upsilon \nabla ^2\vec {u}+{\vec {f}}_b \end{aligned}$$

where $\rho$, $\vec {u}$, $\upsilon$, and *P* are the density, velocity, kinematic viscosity, and static pressure, respectively, and $\vec {f}_b$ is the body force per unit volume in $N/(m^3)$. In Eq. 33, the volume body force vector components created by plasmonic actuation are added to the momentum equation’s right-hand side (Eq. 35). The finite element method, with the Galerkin method, is used to solve these equations and simulate fluid flow with direct interaction with the electrostatic field. The algorithm code is written in the C++ programming language.

**Figure 2:**
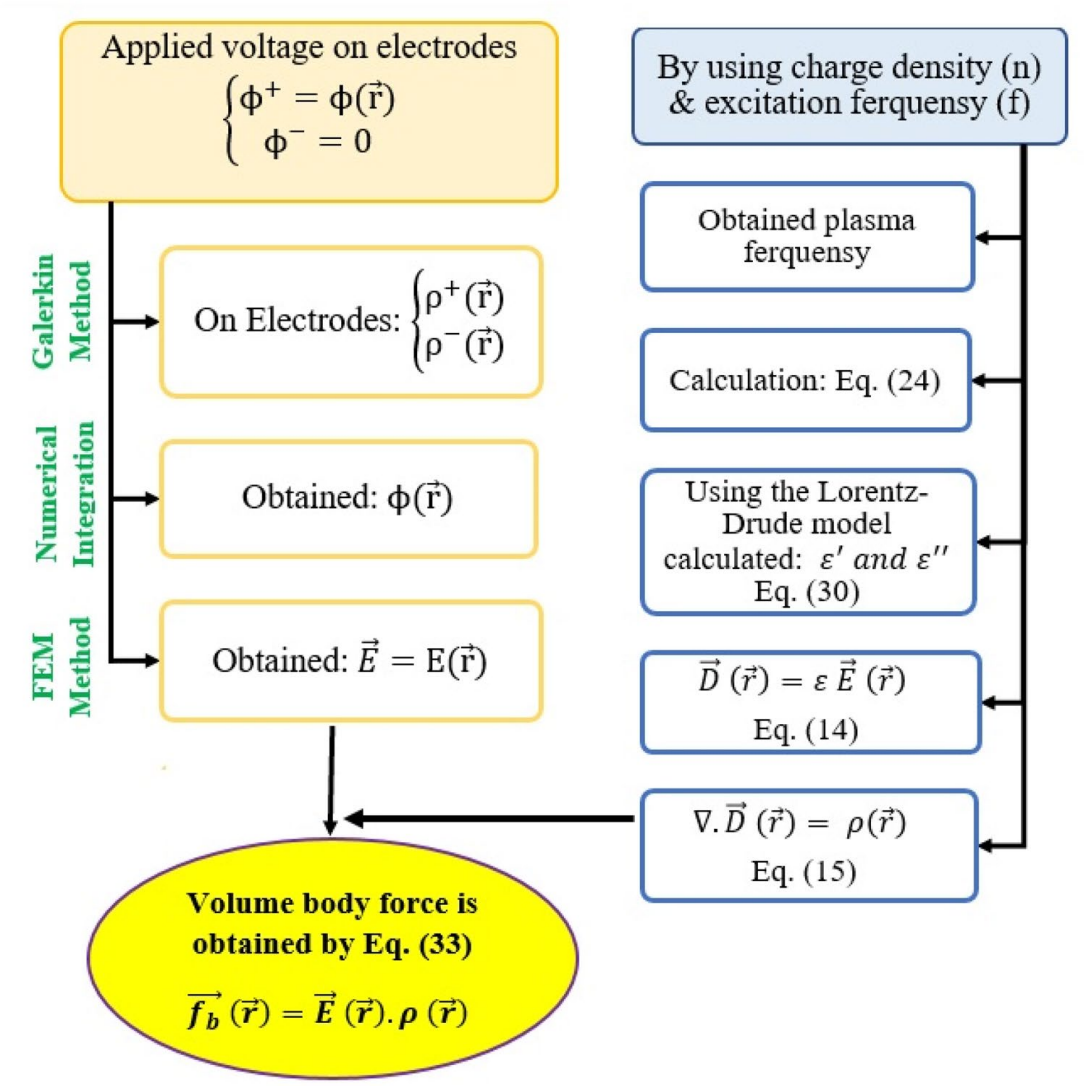
The schematic calculation procedure of the New Lorentz Force (Volumetric Body Force) mathematical procedure with the associated Numerical procedure for each step.

To better explain the numerical implementation procedure in practice, Fig. 2 illustrates a schematic of the mathematical process at each step, as well as the numerical scheme associated with each of the processes. As stated previously, we began modeling from the diagram’s boundary conditions. The voltage between the two electrodes and its excitation frequency are known from the boundary conditions  and initial conditions, respectively. Aside from describing the mathematical procedure described previously, the diagram also specifies the numerical algorithm used in each step. The integral equation that provides the electric charge distribution responsible for the known voltages on the two electrodes was solved using the finite element method with the Galerkin weighting function. The space electric potential field was also calculated using numerical integration of the two electrodes’ electric charge distributions. The finite element method was used again to calculate the gradient of the space electric potential field.

### Model implementation

In order to accurately imitate the influence of the plasma actuator on flow dynamics, the domain addressed to the plasma must have two characteristics: first, it must be compatible with plasma formation physics, and second, it must be self-scalable. When applying any model to an area, the first step is to establish the region’s bounds and extension so that the working domain is completely recognized. This study considers a rectangle associated with the area surrounding the DBD actuator’s structure, with precisely determined dimensions to make the region frequency responsive, guaranteeing the compatibility of the model with the physics as well as the self-scalability of the working region.

When compared to a simple parallel plane structure, the existence of a dielectric between the electrodes and the asymmetry results in a qualitatively different pattern of electric field lines in an asymmetric actuator configuration. The electric field is most intense near the inner margins of the two electrodes. As a result, plasma generation is more likely in areas with stronger electric fields. In this layout, the anode surface is the dielectric rather than the electrode, resulting in a pseudo anode. In this regard, the anode region directly adjacent to the pseudo anode is an electron-rich zone. Since the dielectric, in contrast to the cathode, prohibits charge mobility on its surface, we have a concentration of charges on the cathode and a comparatively sparse distribution of electrons on the pseudo anode^26^.

From the above discussion, one understands that the width of the plasma region consists of the whole length of the dielectric layer, or more accurately, the pseudo anode, and a portion of the exposed electrode that needs further discussion to be fully determined.

The Debye length is another length that contributes to the plasmonic area as a general characteristic length scale for plasma discharges. The Debye length, defined as the ratio of the electron thermal velocity divided by the plasma frequency, is a characteristic distance over which ions and electrons can be separated in a plasma^45^. Hence, a plasma plum will form a sphere with the radius of the Debye length in an unbounded space. However, in a region bounded by the structure of DBD actuator, we can expect the plasma region to fill a semi-sphere, with the radius of the Debye length and the origin at the trailing edge of the exposed electrode.

**Figure 3:**
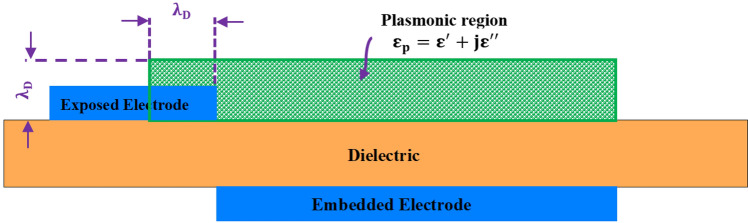
The schematic of a DBD plasma actuator to illustrate the defined plasmonic region by the presented model.

Based on the foregoing, the domain of the plasmonic area is estimated as a rectangle with a height equal to the Debye length and a width beginning at a portion of the exposed electrode equal to the Debye length and ending at the trailing edge of the embedded electrode. Figure 3 illustrates the described plasmonic region. This region provides the self-scalability for the model. It should be noted that the the plasma is assumed to have constant permittivity for the entire domain for the sake of simplicity. The more accurate distribution of the permittivity, as well as its dependency on the applying voltage and the exciting frequency, is going to be undertaken in future research. The Debye length $\lambda _D$ is given from the following empirical relation^34,35^:

36 $$\begin{aligned} \lambda _D\left( m\right)&=0.2\left( 0.5611\times {tan}^{-1}\left( -170.3\times {f\left( kHz\right) }^{-5.124}\right) +1.768\right) \nonumber \\&\quad \times \left( 0.3\times {10}^{-3}\times V_{app}\left( kV\right) -7.42\times {10}^{-4}\right) \end{aligned}$$

It is clear that based on the previous equation, the Debye length changes due to the change in the applied voltage and the exciting frequency. Since the area considered as the plasmonic region is defined based on the Debye length, this area will change as a consequence of changes in the voltage and frequency.

### Model limitations

Intensive work has been done to model the fluid flow impacted by the plasma actuators as accurately as possible while keeping the computational cost near the optimum. These models give basic algorithms for simulating plasma actuators. However, they disregard the intricacies of plasma creation, modeling the integral effects of the plasma jet on the flow to avoid the complexity and high computational expense of a proper investigation of the physical phenomena. Furthermore, these models rely on experimental data to change their characteristic properties. Various formulations have been offered in the literature^23,26–28,31,35,36,38,39^. These formulations have become optimized and applicable yet add to the complexity of the model, and still avoid the implementation of the plasma physics. Details of plasma dynamics have been implemented using the provided approach to establish a criterion for the plasma to generate fluid flow while keeping the model simple and low in computational cost compared to fundamental principle-based models. Nonetheless, the proposed model is currently being refined to provide complete controllability over the actuating body force components. Furthermore, the model is restricted to the frequency (1–14kHz), and the applied voltage (3k–20kVpp) ranges. While experience has shown that the ranges are adequate for most engineering applications, more precise reasoning will be pursued in future studies.

### Boundary condition

**Figure 4:**
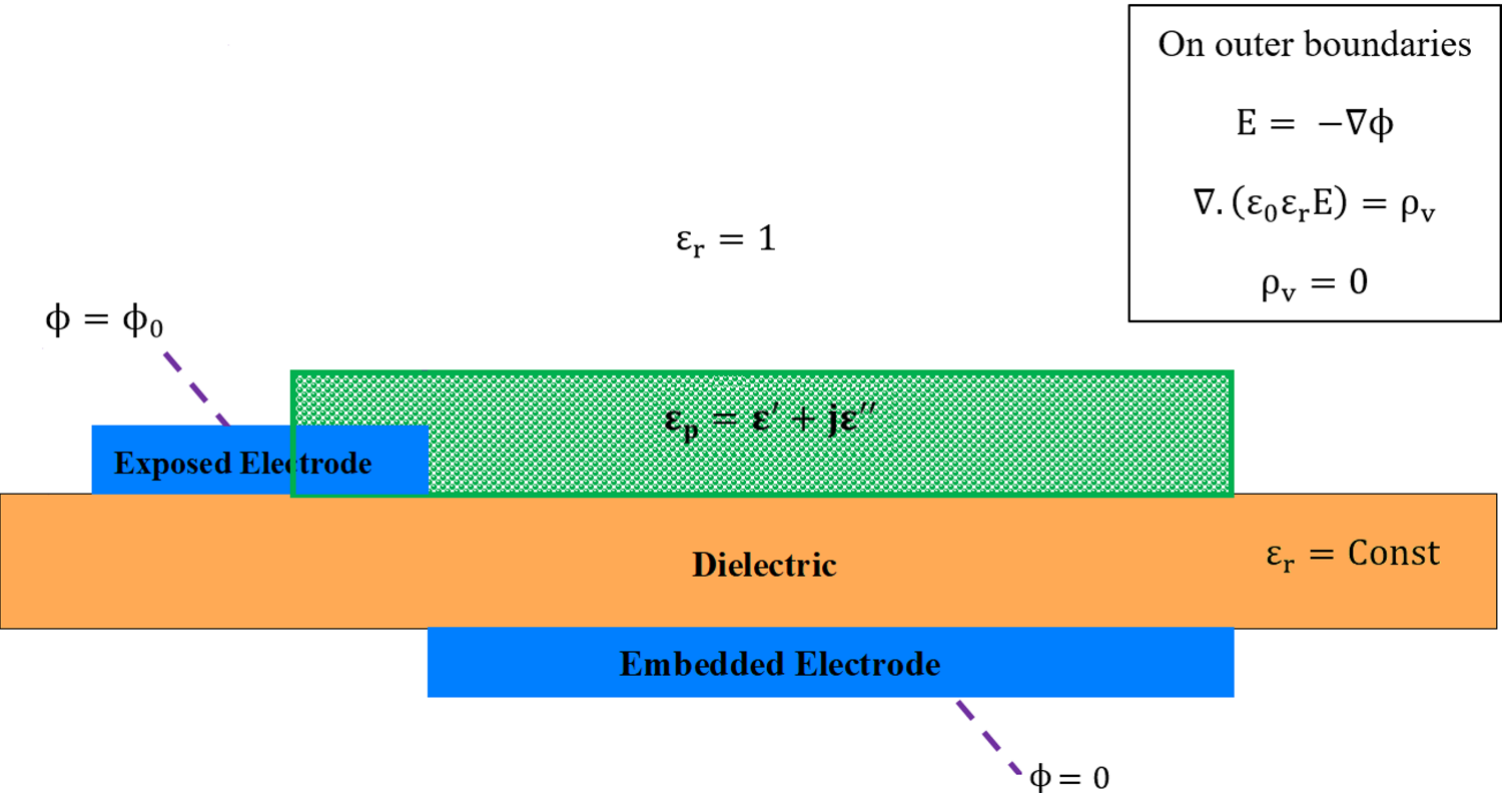
A DBD actuator schematic including the boundary conditions for electrostatic simulation.

Figure 4 illustrates the boundary conditions for the solution of the Poisson equation. The electric potential equation and the Gauss Law’s equation (Eq. 15) is solved in the outer domains and the outer boundaries. As mentioned earlier, the equations and the voltage boundary conditions are modified to be time-independent; therefore, $\phi \ =\ 0$ is set on the embedded electrode, and $\phi \ =\ \phi _{max}/\sqrt{2}\ =\ \phi _{rms}$, on the exposed electrode. $\phi _{max}$ refers to the amplitude of the applied AC voltage.

## Results and discussion

An experiment has been conducted to fully explore the performance of the presented model on predicting the effects of a plasma actuator on fluid flow. Furthermore, to analyze the applicability and adjustability of the model, the experimental case from Kotsonis et al.^46^ has been selected for the study. A final comparison was made based on an experiment by Palmeiro et al.^47^ regarding nominated numerical modeling cases with different actuator configurations and applied voltages and excitation frequencies.

These studies provide a comprehensive understanding of the applicability of the presented model based on the experimental data and a thorough comparison concerning other numerical approaches. The reports presented in the following are based on simulations to ensure the validity of the discussion.

For simulation purposes, the reference geometry of the DBD configuration varies depending on the chosen situations. These geometries are considered 2-D shapes for the simulation, which is a reasonable assumption given the considerable length to thickness ratio of all DBD combinations. The meshing was with triangular pieces, and the mesh setting was initially set to “extremely fine.” The grid spacing was limited to no more than the Debye length. Furthermore, an adaptive mesh refinement was used to achieve grid independence of the acquired results while also minimizing the numerical cost for the simulation of each case to provide perfection with the meshing based on the multiphysics nature of each problem. Table 2 contains the test conditions and experimental data for all of the conducted experiments as well as the study cases. In all cases, the working fluid is air at standard condition ($\nu = 1.75e5 m^{2} s^{-2}$ and $\rho = 1.18 kgm^{-3}$).

**Table 2:** The nominal experimental conditions and parameters as well as specifications and conditions for the study cases.

Specifications	Experiment	Kotsonis et al.^46^	Palmeiro et al.^47^		
			Case A	Case B	Case C
Exposed electrode length (lex)	10 mm	10 mm	6.35 mm	12.7 mm	5 mm
Embedded electrode length (lem)	30 mm	10 mm	6.35 mm	12.7 mm	5 mm
Electrode gap (lg)	0 mm	0 mm	1 mm	1 mm	0 mm
Electrode thickness (te)	0.05 mm	0.06 mm	0.074 mm	0.074 mm	0.074 mm
Dielectric thickness (td)	0.6 mm	0.11 mm	0.19 mm	0.57 mm	0.18 mm
Dielectric constant	2.7	2.7	2.9	2.9	2.9

### Results from conducted experiments

**Figure 5:**
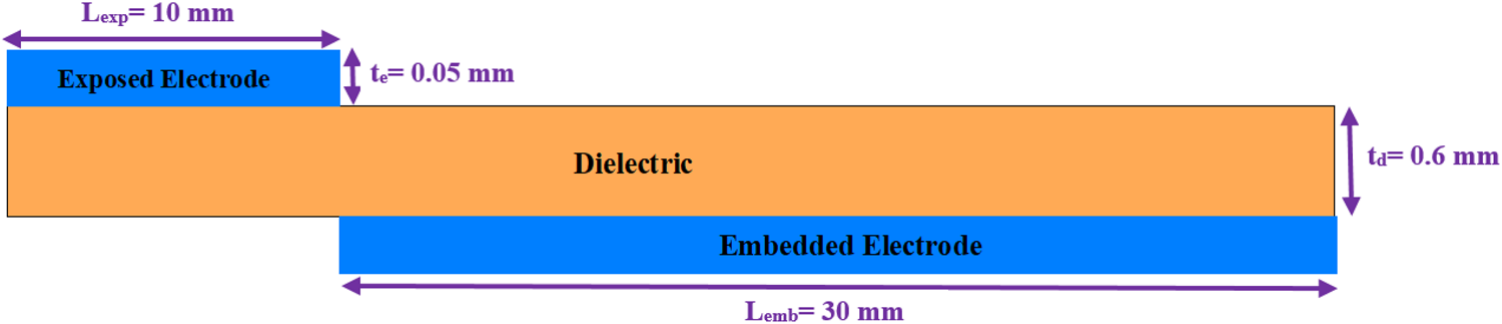
The actuator arrangement utilized in the studies is depicted schematically.

The obtained results from the conducted experiments are presented in this section. Details regarding the experimental setup and measurement systems are discussed in the section titled “Method” section. Figure 5 provides the configuration used for the experiment. The velocity profiles are the metric used to assess the efficacy of the proposed model in predicting the strength of the simulated induced jet and its interaction with the neighboring fluid in comparison to the experimental data. The Applied voltages and frequencies for the experiments and the corresponding simulations are 6,7.2 kVpp and 6, 8 kHz, respectively. As mentioned in Table 2 the length of the exposed and embedded electrodes are 10 mm and 30 mm, respectively. The thickness of the electrodes is 0.05 mm, and the dielectric is made up of polyimide Kapton tape with a total thickness (including adhesive layer) of 0.6 mm and relative permittivity of 2.7.

For this scenario, the computing domain is a rectangle 104 cm long and 50 cm high. The bottom boundary condition is set to no-slip, and the upper boundary is subject to the symmetry criterion. The velocity at the left inlet boundary is set to be zero, and the pressure at the right outlet boundary is set to be zero, as well. The actuator is adjusted at around 30% near the inflow boundary.

**Figure 6:**
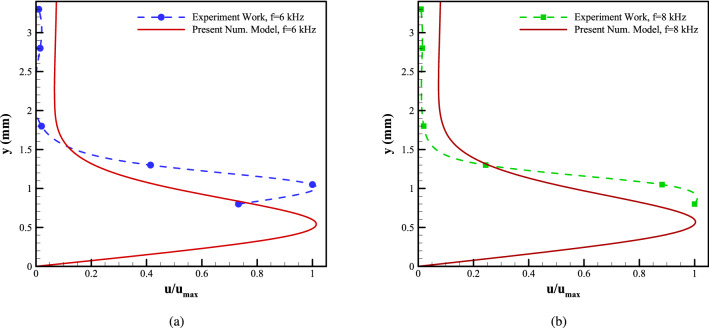
Comparison of velocity profiles from the experiments and the presented numerical scheme for **a** V = 6 kVpp, f = 6 kHz, and **b** V = 6 kVpp, f = 8 kHz, at 5 mm downstream of the exposed electrode’s leading edge.

Figure 6, represent the induced velocity profiles obtained using the current model, compared to the experimental data at a station 5 mm downstream of the exposed electrode’s leading edge for the applied voltages of 6 kVpp and the excitation frequency of 6, 8 kHz, respectively. Based on the flow characteristics, the Debye length (36) is calculated to be 0.114 mm and 0.118 mm, respectively. It is observed that although the presented numerical model underestimates the scaled velocity, it is capable of capturing the general trend of the velocity profile. Moreover, the model estimates the height where the maximum velocity occurs, characterizing the jet-induced boundary layer thickness with acceptable accuracy.

**Figure 7:**
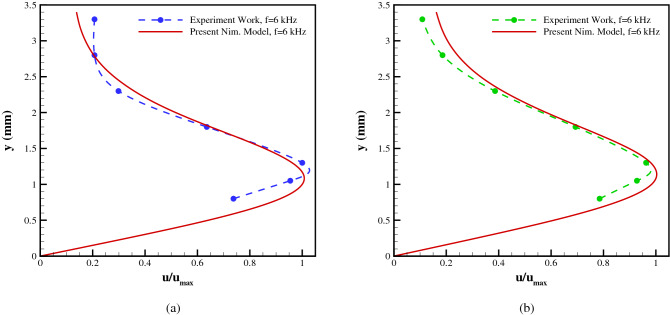
Comparison of velocity profiles from the experiments and the presented numerical scheme for (**a**) V = 6 kVpp, f = 6 kHz, and (**b**) V = 6 kVpp, f = 8 kHz, at 12.5 mm downstream of the exposed electrode’s leading edge.

Figure 7, also, represent the induced velocity profiles obtained using the current model, compared to the experimental data at a station 12.5 mm downstream of the exposed electrode’s leading edge for the applied voltages of 6 kVpp and the excitation frequency of 6, 8 kHz, respectively. It is observed that the presented numerical model is capable of capturing the general trend of the velocity profile. Moreover, the model estimates the height where the maximum velocity occurs, characterizing the jet-induced boundary layer thickness with acceptable accuracy.

**Figure 8:**
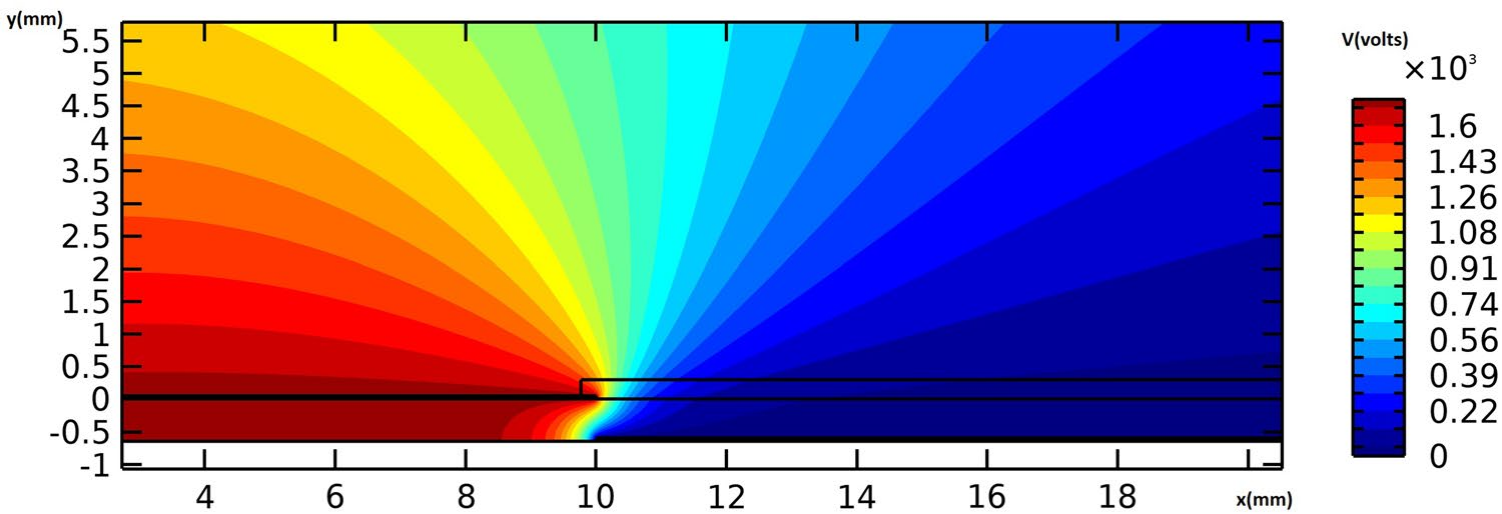
The electric potential field around the actuator in volts, for the actuation case of V = 6 kVpp and f = 8 kHz.

**Figure 9:**
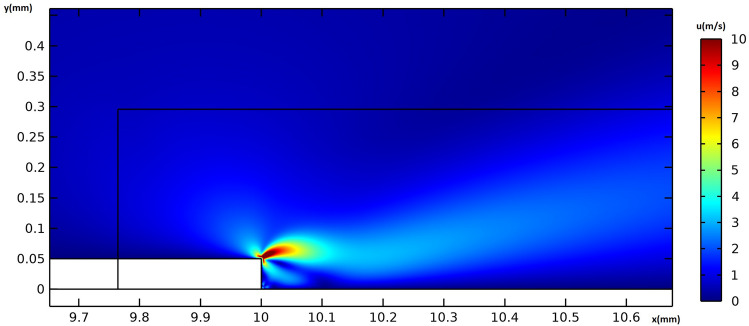
The flow velocity field around the actuator in $ms^{-1}$, for the actuation case of V = 6 kVpp and f = 8 kHz.

Figure 8 provides the electric potential field around the actuator. The maximum voltage difference is observed between the edges of the embedded and exposed electrodes, resulting in the maximum electric field magnitude. Figure 9 provides the flow velocity field around the vicinity of the actuator, indicating that the majority of momentum increases occur in the x-direction. A weak suction effect is also found upstream of the inner edge of the electrodes, indicating the presence of a potentially large pressure differential near the actuator.

**Figure 10:**
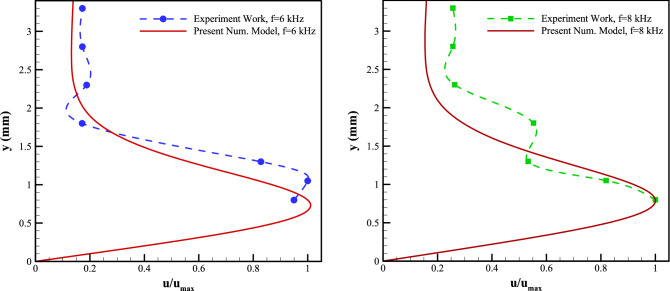
Comparison of velocity profiles from the experiments and the presented numerical scheme for (**a**) V = 7.2 kVpp, f = 6 kHz, and (**b**) V = 7.2 kVpp, f = 8 kHz, at 5 mm downstream of the exposed electrode’s leading edge.

Figure 10 provides the induced velocity profiles for the applied voltages of 7.2 kVpp and the excitation frequency of 6, 8 kHz, respectively, comparing the current model to the experimental results at a station 5 mm downstream of the exposed electrode’s leading edge. Based on the flow characteristics, the Debye length (36) is calculated to be 0.0228 mm and 0.0229 mm, respectively. The presented results show that the numerical model is able to accurately simulate the general trend of the velocity profile as well as predict the height where the maximum velocity occurs.

**Figure 11:**
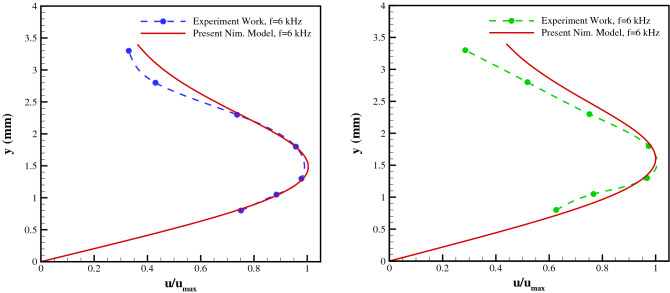
Comparison of velocity profiles from the experiments and the presented numerical scheme for (**a**) V = 7.2 kVpp, f = 6 kHz, and (**b**) V = 7.2 kVpp, f = 8 kHz, at 12.5 mm downstream of the exposed electrode’s leading edge.

Moreover, Fig. 11 provides the induced velocity profiles for the applied voltages of 7.2 kVpp and the excitation frequency of 6, 8 kHz, respectively, comparing the current model to the experimental results at a station 12.5 mm downstream of the exposed electrode’s leading edge. The presented results show that the numerical model is able to accurately simulate the general trend of the velocity profile as well as predict the height where the maximum velocity occurs.

**Figure 12:**
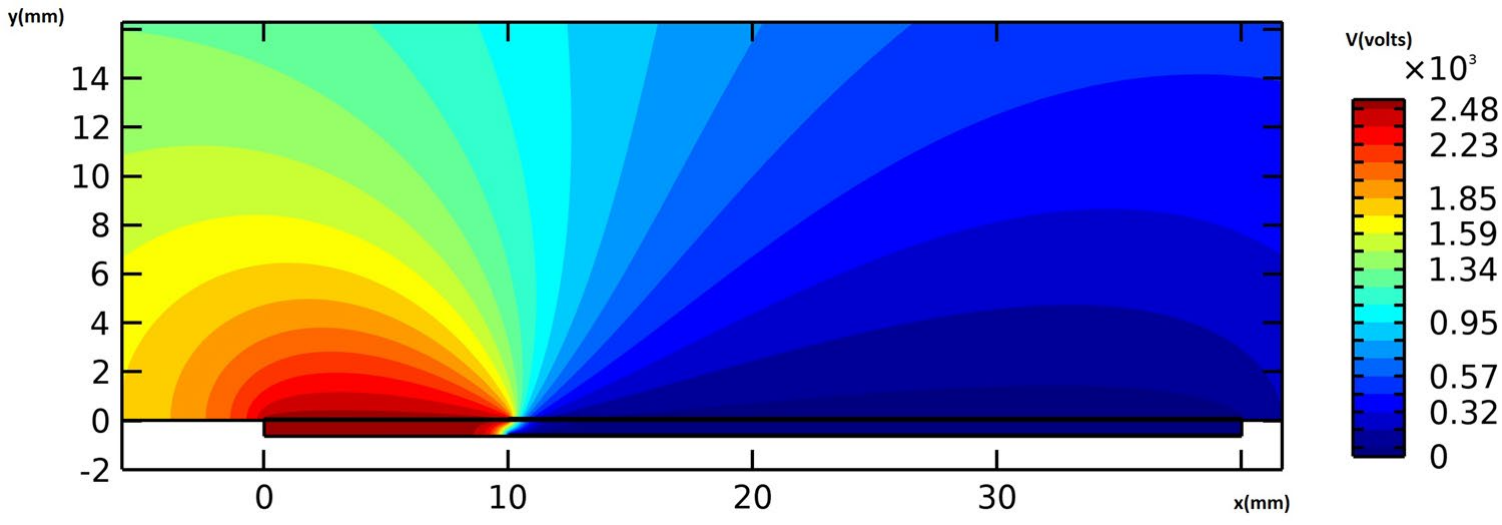
The electric potential field around the actuator in volts, for the actuation case of V = 7.2 kVpp and f = 8 kHz.

**Figure 13:**
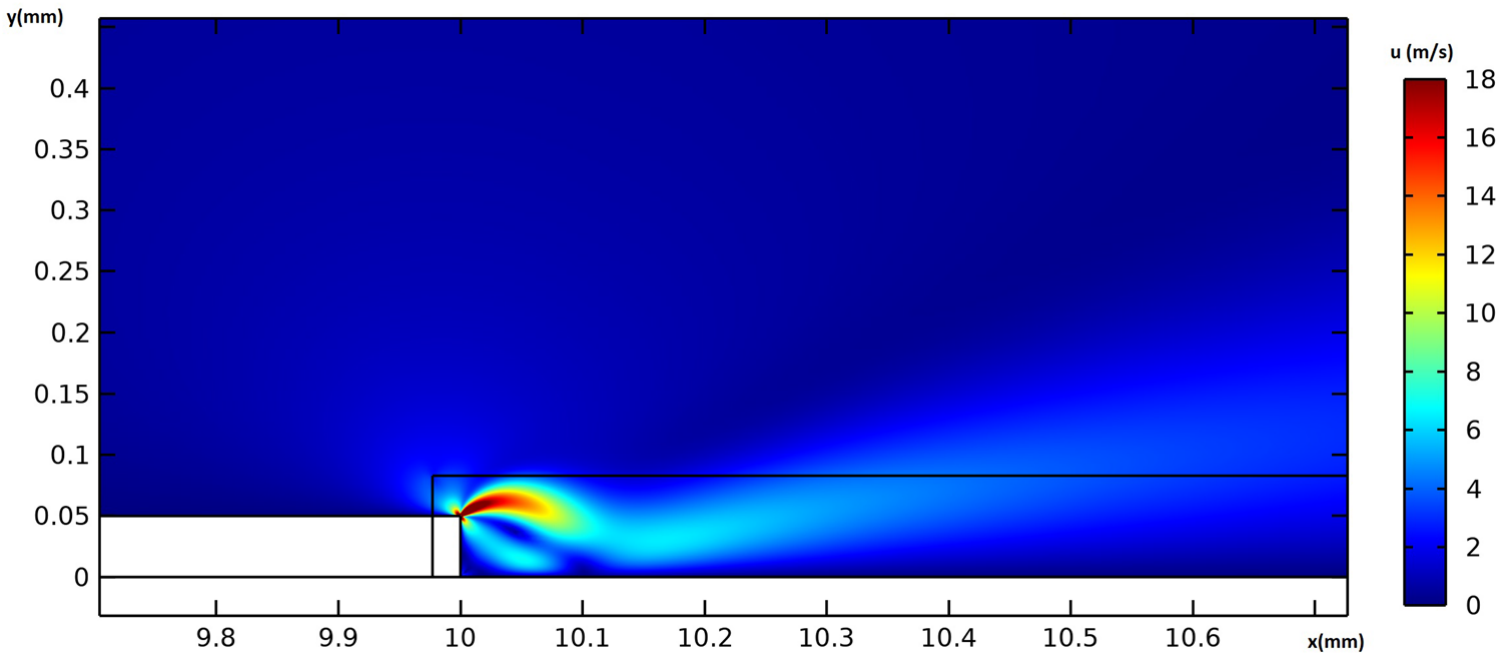
The flow velocity field around the actuator in $ms^{-1}$, for the actuation case of V = 7.2 kVpp and f = 8 kHz.

Same as the previous cases, Fig. 12 provides the electric potential field around the actuator. It is observed that the maximum voltage difference occurs between the edge of the embedded and exposed electrodes resulting in the maximum electric field magnitude. Figure 13 represents the flow velocity field around the vicinity of the actuator, showing that the momentum increases mainly in the x-direction, and a potentially strong pressure gradient due to a weak suction effect exists upstream the inner edge of the electrodes.

The maximum velocities obtained using the presented numerical model and the corresponding results from the experiments are tabulated in Table 3, for voltages 6, and 7.2 KVapp and frequencies 6, and 8 KHz, respectively, at two distances along the x-direction, 5 mm and 12.5 mm downstream of the exposed electrode’s leading edge. The results reveal that the model predicts the maximum velocity, as an indication of the total body force, with acceptable accuracy.

**Table 3:** The comparison of the calculated integrated horizontal body force component for different input voltages and the experimental results.

	V kV	6 kHz-current model	6 kHz-experiment	8 kHz-current model	8 kHz-experiment
x = 5 mm	6	0.73	0.74	0.71	0.73
	7.2	1.16	1.83	1.03	1.10
x = 12.5 mm	6	0.95	0.98	0.86	0.83
	7.2	1.09	1.19	0.44	0.77

### The experiment of Kotsonis et al

The experiment of Kotsonis et al.^46^ on the body force field of DBD actuators is selected to serve as a benchmark, providing certainty with the model. Kotsonis et al. provide body force fields derived from PIV observations for various input voltages. The details of the plasma actuator configuration used in the work of Kotsonis et al. are presented in Table 2. The plasma actuator configuration used by Kotsonis et al. consists of electrodes with a width of 10 mm and 0.06 mm thickness. The electrodes are separated by a horizontal gap of zero. Furthermore, two dielectric layers of polyimide Kapton tape with a total thickness (including the adhesive layer) of 0.11 mm separate the electrodes. The peak-to-peak voltage on the electrodes was adjusted from 8 to 16 kVpp in 2 kVpp steps. For each input peak-to-peak voltage, the body force field is monitored.

The computational domain and the boundary conditions are set to be the same as in the previous section. Based on the flow characteristics provided by Kotsonis et al. the Debye length is considered to be 2 mm. In this regard, the area considered as the plasmonic region would be a rectangle with the height of the Debye length and a width starting from a portion of the exposed electrode that is equal to the Debye length and ends at the trailing edge of the embedded electrode.

**Figure 14:**
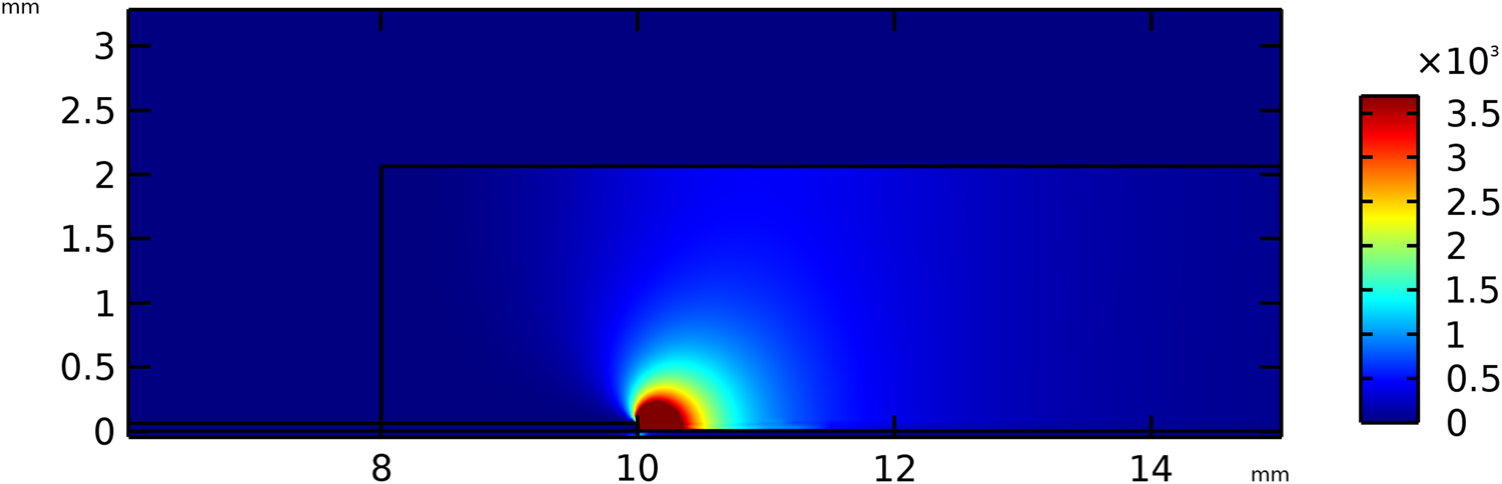
Spatial distribution of the horizontal body force component calculated based on the presented numerical model.

Figure 14 provides the spatial distribution of the body force components based on the presented numerical model. It is observed that the maximum horizontal body force is produced at the edge of two electrodes where, as predicted, is the region with the highest electric field magnitude, therefore, with the most probability for ionization.

Table 4 compares the computed integrated horizontal body force component to the experimental results of Kotsonis et al. for various input voltages, respectively. A comparison is made based on the integrated body forces on the area surrounding the plasmonic region. The Kotsonis et al. experiment area was a rectangle with the height of the Debye length’s order and a width starting from 10% of the exposed electrode and ends at 70% from the trailing edge of the embedded electrode. One may interpret the results by saying that the presented model is capable of accurately predicting the integral effect of the body force production. The resultant body forces are accurately predicted with a maximum deviation of 7.69% from the experimental results.

According to the literature, the generated thrust should increase by $V^{7/2}$^32^. This proportionality also applies to the total body force generated, $f_b\propto V^{7/2}$. The results from the numerical simulation reveals that the obtained integrated body forces agree with the proportionality found in literature.

**Table 4:** The comparison of the calculated integrated horizontal body force component for different input voltages and the experimental results.

Vmax(kV)	fx (N)-experiment	fx (N)-Numerical	Error (%)
4	0.00006	0.000058	3.33
5	0.0013	0.0012	7.69
6	0.0032	0.0031	3.13
7	0.0051	0.005	1.96
8	0.0093	0.0089	4.30

### The work of Palmeiro et al

**Figure 15:**
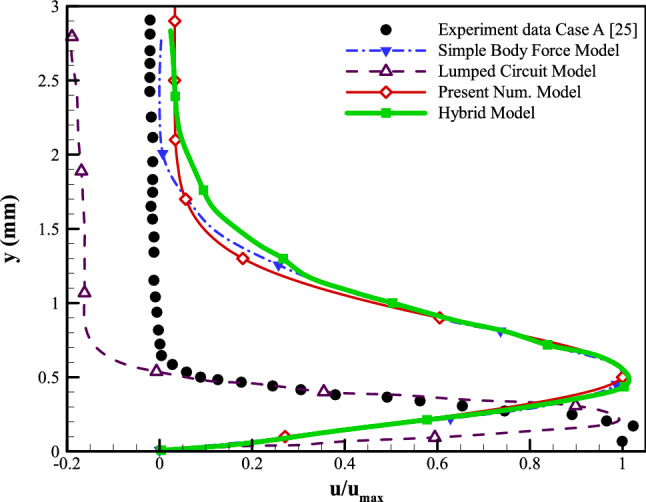
The results from the presented numerical model compared to the experimental and other numerical vertical velocity profiles on actuator geometry of Case A.

**Figure 16:**
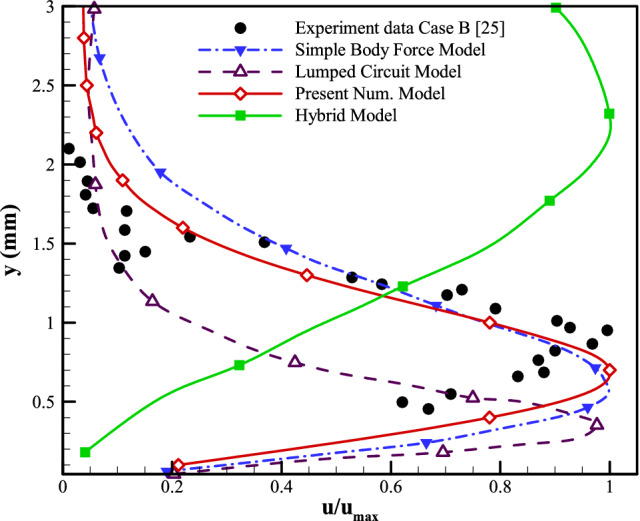
The results from the presented numerical model compared to the experimental and other numerical vertical velocity profiles on actuator geometry of Case B.

**Figure 17:**
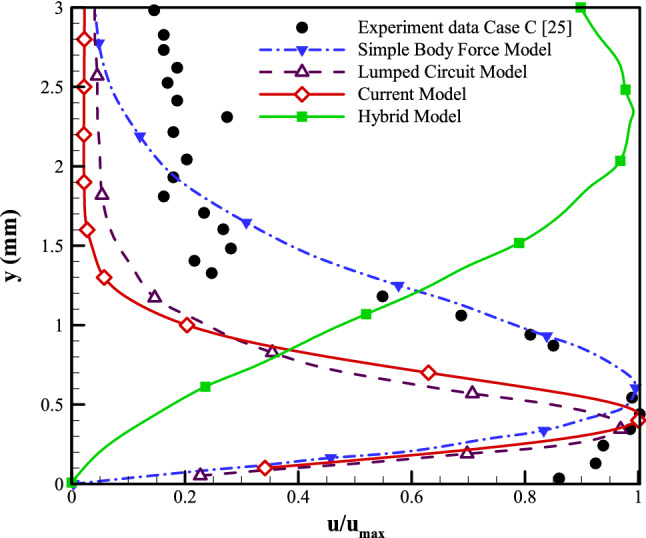
The results from the presented numerical model compared to the experimental and other numerical vertical velocity profiles on actuator geometry of Case C.

The final case to examine the applicability of the presented modeling strategy has been selected to be the experimental work of Palmeiro et al.^47^. Furthermore, we compare the current model to various numerical models using the numerical works offered by Palmeiro et al. Following his studies, three test scenarios are explored. The details of all the cases are given in Table 2. For each test case, five sets of results are presented: (A) the experiment^47^; (B) the lumped-circuit model^25^; (C) the hybrid model^24^; (D) the simple body force model^26^, and (E) the current numerical model. The corresponding maximum velocity normalizes the velocity profiles for every modeling methods. As tabulated in Table 2, the plasma actuator configuration used for cases A–C of Palmeiro et al. consists of electrodes with widths of 6.35, 12.7, and 5 mm, respectively, all with a thickness of 0.075 mm. The horizontal gap used between the electrodes is set to be 1 mm, 1 mm, and zero, respectively. The electrodes, in all cases, are separated by a dielectric layer of polyimide Kapton tape with a total thickness of 0.19, 0.57, and 0.18 mm, respectively. Moreover, the input peak-to-peak voltage on the electrodes is 12, 15, and 10 kVpp, respectively, with excitation frequencies of 3, 3, and 2.75 kHz, respectively. The computational domain, as well as the boundary conditions, are set to be the same as in previous sections. Based on the flow characteristics, the Debye length (36) for cases A–C is calculated to be 0.46, 0.74, and 0.28 mm, respectively. In this regard, the area considered as the plasmonic region would be a rectangle with the height of the Debye length and a width starting from a portion of the exposed electrode equal to the Debye length and ends at the trailing edge of the embedded electrode. Figures  15, 16 and 17 provide the induced velocity profiles obtained based on the experimental and the numerical results from Palmeiro et al. compared to the results from the presented numerical model for cases A, B, and C, respectively.

Based on the results for case A (Fig. 15), the presented numerical model performs as well as the Simple Body Force and the Hybrid models in estimating the velocity profile. While the three models over-predict the normalized velocity, the presented model provides the best result. The Lumped Circuit model is the one method that correctly predicts the normalized velocity while it deviates from the experimental results when moving away from the actuator.

Comparing the numerical results to the experimental data in Fig. 16 for case B, it is observed that the presented numerical model provides the best prediction of the velocity profile compared to the other numerical schemes. Both the presented numerical model and the Simple Body Force model can accurately predict the jet boundary layer thickness, while the results of the latter deviate from the experimental data as we move away from the actuator. The figure shows that the Hybrid model fails to capture the velocity profile, and the Lumped Circuit model underestimates the normalized velocity although capturing the general trend.

Results from case C (Fig. 17) show that the presented numerical model provides the best prediction for the jet boundary layer thickness characterized by the location where the maximum velocity occurs compared to the Simple Body Force and the Lumped Circuit models. However, similar to the Lumped Circuit model, the presented numerical model underestimates the normalized velocity when moving away from the actuator. Unlike the hybrid model, the presented numerical model performs as well as the Simple Body Force and the Lumped Circuit models in capturing the general trend of the velocity profile.

Once the findings from all three examples are reviewed, it is clear that the given modeling method has the most consistent predictive capability for the various test cases compared to the other phenomenological models.

To compare the potentiality of the presented model to other numerical methods more in detail, Table 5 offers the maximum velocity obtained at x = 25 mm from the exposed electrode’s leading edge, respectively, for cases A, B, and C. The present model shows the most consistent predictive capability and adequate accuracy for all three cases in comparison to other numerical scenarios.

**Table 5:** The computed maximum velocity achieved at x = 25 mm from the leading edge of the exposed electrode, compared to numerical technique outcomes for three cases of A, B, and C.

Case study	Experiment^47^	Lumped-circuit model^25^	Hybrid model^24^	Simple body force model^26^	Current model
Case A	1.9458	8.9311	1.5339	0.7266	2.7141
Case B	1.2772	7.3674	0.2927	0.8383	2.3714
Case C	1.5181	3.977	1.2382	0.3042	5.0699

## Conclusion

In this study, a new modeling approach has been outlined for the numerical investigation of the fluid flow affected by a plasma actuator. In this new approach, we presented a model that simulates the plasmonic region based on the practical material model, the Lorentz model, to characterize the region as a dispersive medium. The dissipated energy added to the flow is calculated in terms of local body force components by solving Poisson’s equation for the electric field and implementing the simplified Lorentz model for the polarization field. The area considered as the plasmonic region is defined to be based on the characterizing Debye length, changing as a function of the excitation frequency and the applied voltage. In this regard, the current approach establishes a criterion for the plasma to induce the fluid flow by considering the details of the plasma dynamics. This facilitated us to define the characterizing parameters of the actuation to be self-adjustable based on the physics while keeping the model simple and of low computational cost compared to the fundamental principle-based models. We conducted an experiment to compare the observed influence of plasma actuators on fluid flow with the results predicted by the model to assess the proposed model’s validity and performance. The results indicated that the model could capture the general trend of the velocity profile and estimate the jet-induced boundary layer thickness with acceptable accuracy. The model also estimated the significant increase in momentum in the x-direction regarding the actuator and a potentially significant pressure differential near the actuator. Furthermore, the model’s universality was validated using diverse experiments and exempted numerical models. The integral effects of plasma actuation have been predicted with a maximum error of roughly 8%. The model’s ability to capture the velocity profile, estimate the jet-induced boundary layer thickness, and calculate the induced jet strength was evaluated using experimental data and results from the selected numerical models. The present model shows the most consistent predictive capability and adequate accuracy for all test cases in comparison to other numerical scenarios. The results show that the proposed model and technique have a bright future in plasma flow control applications.

## Methods

**Figure 18:**
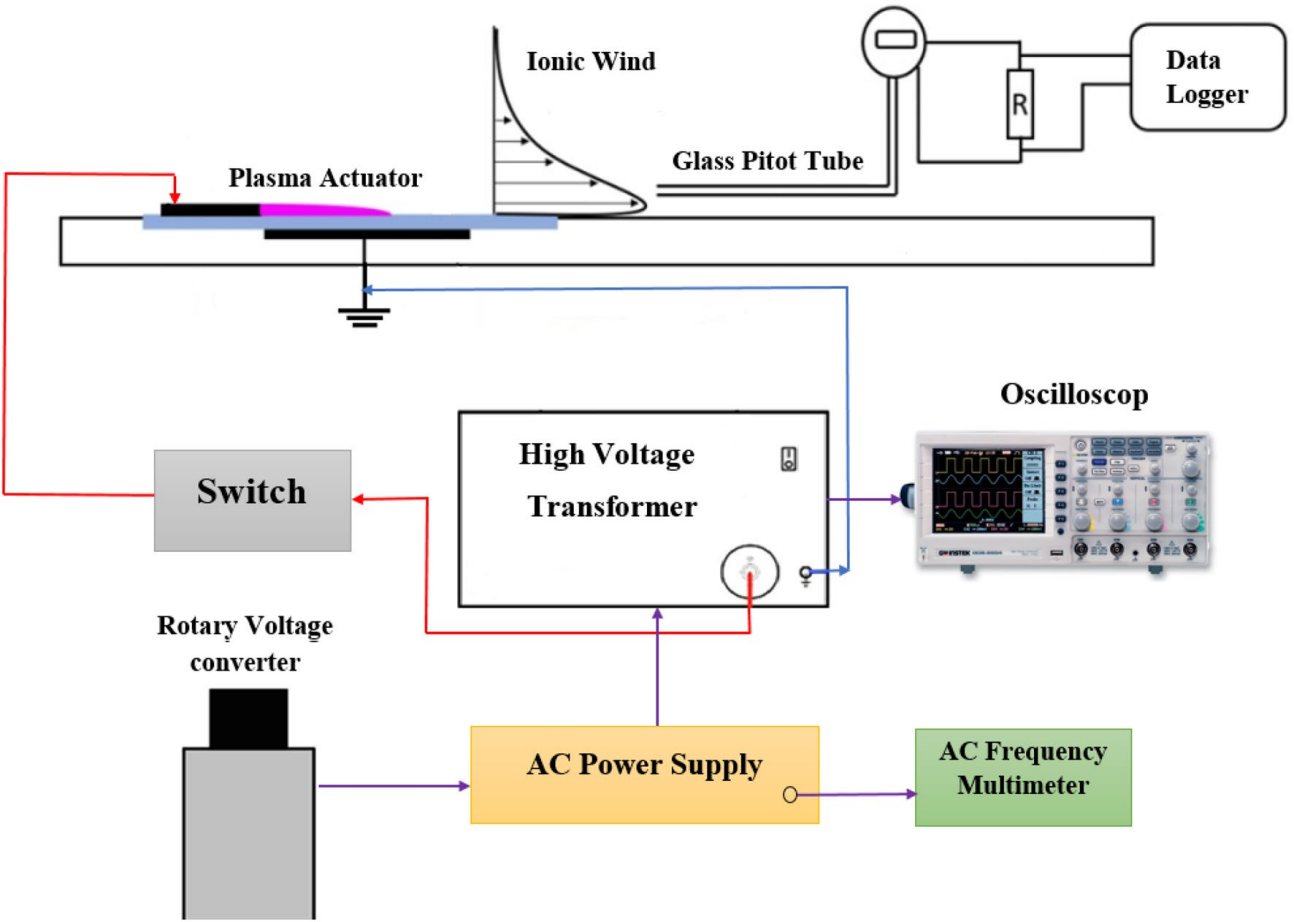
Schematic representation of the experimental setup of the conducted experiment.

The experimental setup and the plasma actuator investigated in this study are illustrated in Fig. 18. The plasma device is comprised of two 0.05 mm thick aluminum electrodes and six layers of Kapton film as a dielectric. The exposed electrode and embedded electrode have widths of 10 mm and 30 mm, respectively. In the streamwise direction, the spacing between the upper and lower electrodes is adjusted to zero. Each actuator is 0.4 m long, and velocity field measurements were performed along the actuator device’s centerline. Measurements were obtained using a 1.6 mm outer diameter glass pitot tube in quiescent air. The probe was directed to measure the streamwise velocity and mounted to a vertical traverse with a resolution of 0.01 mm. Each velocity data point was averaged over a sample measured at 5 kHz for an interval of 10 s. The sample population was recorded using a NI-USB5239 data acquisition device connected to a PC. The downstream location of the measurement relative to the trailing edge of the exposed electrode for each actuator is set to be 5 mm and 12.5 mm respectively, for two study cases. Each excitation signal was sinusoidal and was delivered using a Rigol DG1011 waveform generator. The output sine wave voltage and frequency range of the power supply were, respectively, 0–15 kV and 0–15 kHz. The steady-state wave was used in all of the experiments.
